# Internalizing Symptoms among Kosovar Adolescents: Pubertal Correlates in Boys and Girls

**DOI:** 10.1007/s40653-024-00610-z

**Published:** 2024-02-16

**Authors:** Elona Krasniqi, Alexander T. Vazsonyi, Panajotis Cakirpaloglu

**Affiliations:** 1 Department of Psychology Vodární 6, Palacký University Olomouc, 779 00 Olomouc, Czech Republic; 2 Department of Family Sciences, 316 Funkhouser Building, University of Kentucky, Lexington, Kentucky 40506 USA

**Keywords:** Anxiety, Depression, Somatic complaints, Body mass index, Pubertal development, And sex

## Abstract

Pubertal status/stage of maturation and pubertal timing have been linked with emotional symptoms of problems among youth, particularly in vulnerable developmental contexts at risk for stress exposure. The present study tested the extent to which pubertal status/stage of maturation and pubertal timing were associated with anxious/depressed, withdrawn/depressed, and somatic complaint symptoms in Kosovar adolescents. It also tested whether sex moderated these relationships. Data were collected from N = 1,342 Kosovar adolescents (665 girls; M age = 13.26 years, SD = 1.27; 677 boys M age = 13.19 years, SD = 1.31). Regression analyses provided evidence that pubertal status/stage was positively associated with rates of anxious/depressed, withdrawn/depressed, and somatic complaint symptoms in girls, but only with withdrawn/depressed symptoms in boys. Additionally, pubertal timing was positively associated with anxious/depressed, and somatic complaint symptoms in girls; no significant links were found for boys. The present study provided evidence that advanced pubertal status/stage as well as timing is positively associated with internalizing symptoms in girls; however, only pubertal status/stage was positively associated with withdrawn/depressed symptoms in boys. The study highlights the importance of pubertal development for internalizing symptoms in a developmental context known for high stress exposure, particularly for girls.

## Introduction

One in seven adolescents (14%), ages 10 to 19 years of age, experiences a mental health problem, such as anxiety or depressive symptoms, also expected to be present later in life (Venkatesan, 2023). Research has shown that these problems peak during middle adolescence years around the age 13, particularly among the girls (Angold et al., 1998; Ge et al., 1994; Hankin et al., 1998). It has also shown that developmental changes, particularly in early adolescence, impact an adolescent’s emotional adjustment (Susman & Dorn, 2013). More specifically, *pubertal status/stage* (Ge et al., 1994, 2006; Lewis et al., 2018)*,* the degree of physical maturation of the current morphological development, as well as *pubertal timing* (Deardorff et al., 2021; Hoyt et al., 2020; Ullsperger & Nikolas, 2017)*,* maturational onset relative to same age peers (Susman & Dorn, 2013), have been found to be positively associated with emotional adjustment difficulties. This latter evidence has been found to a greater extent among girls than boys (Deardorff et al., 2007; Patton et al., 2008; Stumper & Alloy, 2023). Other work has shown that timing asynchrony was associated with depressive symptoms among girls, less do for boys (Stumper et al., 2020). Finally, historical data have provided consistent evidence of a downward trend of age of pubertal onset (Eckert-Lind et al., 2020; Mul et al., 2001), thus increasing the urgency to better understand the puberty-internalizing symptoms links. The present study was particularly interested in better understanding these links among Kosovar youth.

The social and cultural environment where adolescents develop might further uniquely contribute to when puberty occurs as well as how it impacts internalizing symptoms. For instance, an earlier onset of puberty has also been found in youth growing up in low SES contexts (Oelkers et al., 2021), thus highlighting the importance of considering SES for understanding pubertal development. Furthermore, other work has shown that growing up in a disadvantaged low SES context is also positively associated with internalizing symptoms (Leventhal & Brooks-Gunn, 2000). Historically, Kosovar youth have been chronically exposed to different collective traumas, which have likely had an impact on their physical and psychological development. The exposure by Kosovar children and adolescents to mass trauma through the Balkan war has been well documented; this work has shown that this exposure has long lasting impacts on physical and mental health, including depressive symptoms (Eytan et al., 2015; Fanaj, 2020). In addition to the exposure to war, these youth have been chronically witnessing ethnic tensions and conflicts, still high today, thus placing them at exceptionally high risk for emotional problems (Shahini & Landsman, 2008). In a post war Kosovo, many family members expressed symptoms of PTSD, faced the loss of family members due to death or simply being missing, and experienced poverty; all of these factors negatively impacted the mental health of children and adolescents (Shahini et al., 2015). Research has provided evidence that 11 years following the end of the war that paternal PTSD was positively associated with children’s anxiety and depressive symptoms (Schick et al., 2013).

Duraku and colleagues (2023) recently found that Kosovar youth born after the war scored higher on measures of PTSD symptoms and lower on a number of perceived support measures (social, significant other, and family), than their parents. This finding was particularly evident in youth who had parents with PTSD in comparison to ones without. These adolescents were more likely to report negative mood and cognition, or emotional arousal; they were also more likely to report experiencing sudden accidental death or sudden violent death in comparison to their peers. This provides evidence of an intergenerational transmission from parents to children of wartime trauma, decades following exposure. In addition, research has also indicated that adolescents from collectivist cultures are more conservative, conforming as well as sensitive about sexual and social norms (Hedge et al., 2022). Thus, this cultural context might also make it less likely for youth to share information about pubertal development/maturation for instance; in other words, Kosovar youth might be likely to talk about puberty in comparison to youth from individualistic cultures like Great Britain or the United States. Therefore, the present investigation sought to better understand the extent to which the unique developmental context of Kosovo might influence the observed links between measures of puberty and internalizing problems.

More specifically, the present study sought to test the extent to which different measures of puberty were associated with measures of internalizing symptoms among Kosovar youth; in addition, it tested the extent to which these links were moderated by sex. The study also tested the extent to which socioeconomic status was associated with pubertal development status/stage, pubertal timing, and measures of internalizing symptoms.

## Body Mass Index, Puberty, and Externalizing Symptoms

Previous research has provided evidence of a high prevalence of overweight ethnic Albanian children and adolescents, living in Kosovo, Albania, FYR Macedonia (North Macedonia) as well Montenegro (Tarp et al., 2018). The underlying mechanisms of the relationship between Body Mass Index and pubertal development are not fully understood, however, research indicates that there is a positive association between pubertal development and higher BMI (Kaplowitz et al., 2001). Girls with a higher BMI are more likely to start their menses earlier (Kaplowitz, 2008), and they also reached breast stage 2 development at younger ages (Biro et al., 2013). Boys with greater BMI also entered puberty at younger ages (Sørensen et al., 2010; Tomova et al., 2015). BMI is associated with internalizing symptoms, although to a greater extent among girls than boys (Ames et al., 2015; Needham & Crosnoe, 2005). Research has also found that heavier than average as well as underweight girls, but also obese boys, report higher levels of depressive symptoms (Cortese et al., 2009) and overall internalizing problems, in comparison to average BMI groups (ter Bogt et al., 2006). Others have found no associations between BMI and depression (Swallen et al., 2005).

## Pubertal Development and Internalizing Problems

Advanced pubertal status/stage has been found to be positively associated with depression (Angold et al., 1998; Lewis et al., 2018; Huerta & Brizuela-Gamiño, 2002), anxiety (Reardon et al., 2009) and somatic symptoms (LeResche et al., 2005; Rhee, 2005) among youth, more so among girls than boys (Marceau et al., 2012). Early pubertal timing also has been associated with depression (Barendse et al., 2022; Deardorff et al., 2007; Hamlat et al., 2020), and anxiety symptoms (Barendse et al., 2022; Senia et al., 2018), in comparison to ones who mature on time or late. In addition, early pubertal timing is also more prevalent in girls in comparison to the boys (Blumenthal et al., 2011; Deardorff et al., 2021) and positively associated with internalizing symptoms in both boys and girls (Ullsperger & Nikolas, 2017). However, Angold et al. (1998) found that pubertal status/stage, rather than pubertal timing, was a significant predictor of depression among adolescent girls (cf. Copeland et al., 2019). Others found similar positive associations between both pubertal status/stage as well as pubertal timing and internalizing symptoms in girls and boys (Ge et al., 2006).

Particular social and environmental circumstances might exacerbate the associations between pubertal status/stage or pubertal timing and externalizing symptoms. For instance, Ge and colleagues (Ge et al., 1994) found evidence that increased environmental stress was uniquely associated with depressive symptoms in girls at age 13, but not boys. Similarly, Ge and colleagues (2001) found that advanced pubertal status/stage girls who had stressful life experiences were more vulnerable to depressive symptoms. Adversities that occur during life such exposure to poverty, might accelerate the pubertal development that in turn is positively associated with internalizing problems.

## The Kosovar Developmental Context

Kosovar girls report elevated levels of internalizing problems in comparison to boys (Fanaj et al., 2015; Jetishi & Muqaj Froku, 2016; Shahini et al., 2015). Estimates of internalizing problems among Kosovar adolescents by Shahini and colleagues (2015) were 25.5% (some of the highest were anxious/depressed (19.4%), withdrawn/depressed (22.1%), and somatic complaints (14.9%)). Considering that Kosovo has been going through tremendous socio-economic and cultural transitions over the past two decades (Latifi, 2014; Shahini et al., 2015; Tawil, 2009; The World Bank, 2017), this calls for research to better understand the extent to which these unique changes have impacted youth emotional adjustment. Post-war Kosovar period of transition has impacted family economic conditions, with unemployment rates of 25.0% (Kosovo Agency of Statistics, 2020). Economic hardship is associated with a whole host of deleterious consequences, including a lack of proper nutrition, and family stressors, which further might potentiate youths’ overall well-being. In addition to that, Kosovo has experienced a profound shift over the past two decades from collectivism to individualism, which has manifested itself in a number of ways, including working independently rather than relying in one’s family income, increased levels of both education and employment among women, and a shift to residing as a nuclear family (parents and children only) rather than living in an extended family (Flere & Klanjšek, 2013; Kadriu, 2018; Shaqiri, 2018). Following the 1998/1999 war in Kosovo, many Kosovar families moved from villages to big cities for better job opportunities and education (Shahini & Landsman, 2008). As such, emotional struggles might further negatively impact youth psychologically. The contextual amplification hypothesis proposed by Ge and Natsuaki (2009) highlights that experiencing early pubertal transitions in a disadvantaged context greatly increases the risk for adjustment difficulties among adolescents. In addition, as suggested by Duraku et al. (2023), the indirect intergenerational transmission of trauma through family members or parents has had and continues to have an impact on the affective state of Kosovar youth until today. Thus, the present study seeks to shed some new light on whether these social, cultural, and economic transitions might have impacted adolescent emotional adjustment among Kosovar boys and girls.

## Theoretical Grounding

Several theories have been developed in an attempt to explain why transitioning through puberty is associated with internalizing problems. Notably, the empirical evidence supports that the association between pubertal development and internalizing symptom links might have some contextual explanation, namely simply where youth reside (Angold et al., 1998; Ge & Natsuaki, 2009; Ge et al., 2001). According to Lewis and colleagues (2018: *pubertal status hypothesis*), puberty simply increases the risk of depression, independent of its timing. Next, *developmental readiness hypothesis* (also known as the stage termination hypothesis), posits that early maturational timing heightens the risk for adolescents to experience internalizing problems, particularly among early maturing girls (Negriff & Susman, 2011; Petersen & Crockett, 1985). Early maturing youth, and particularly girls might have an insufficient level of cognitive maturation to deal with the very novel bodily changes as well as societal behavioral expectations. The asynchrony between cognitive and biological maturity as well as social pressures (positive as well as negative) might be responsible for creating a gap among these three critical components, resulting in increased levels of emotional problems in early maturing adolescents. The *contextual amplification hypothesis* proposes that experiencing early pubertal transitions in a disadvantageous context increases the risk for psychopathology symptoms (Ge & Natsuaki, 2009). Therefore, it was expected that advancing through puberty would be positively predicting rates of internalizing symptoms. In addition, it was expected that early maturing youth would score higher on measures of internalizing symptoms, in comparison to adolescents who mature on time and/or late. These relationships were particularly salient in the present study as Kosovar youth reside in a developmental context characterized by exposure to collective trauma, poverty, ethnic tensions, as well as both undiagnosed and untreated parental mental health problems following the war.

## The Present Study

Given some of the inconsistent evidence as well as gaps in the previous literature, the present study sought to test the extent to which pubertal status/stage and early pubertal timing was associated with anxious/depressed, withdrawn/depressed as well somatic complaints symptoms in youth. At the same time, the tested also tested whether sex differences existed in internalizing problems. The present study extended the work by Shahini and colleagues (2015) as well Fanaj and colleagues (2015), which found that Kosovar girls reported more internalizing problems in comparison to boys. Likewise, the study sought to advance this previous work by focusing on whether advanced pubertal status/stage of maturation or timing of puberty was positively associated with emotional problems in Kosovar youths, novel observation in the Kosovar developmental context.

## The Study was Guided by the Following Research Questions:

1. Is pubertal status/stage positively associated with rates of internalizing problems, namely anxious/depressed symptoms, withdrawn/depressive symptoms, and somatic complaint symptoms in Kosovar adolescents?
2. Is early pubertal timing positively associated with the rates of internalizing problems, namely anxious/depressed symptoms, withdrawn/depressive symptoms, and somatic complaint symptoms in Kosovar adolescents, in comparison with on-time or late maturing youths?
3. Are there sex differences in rates of internalizing symptoms independent of maturational timing and pubertal status/stage of development?

## The Study Tested the Following Hypothesis:

1. It was expected that pubertal status/stage would be positively associated with the rates of anxious/depressed and withdrawn/depressed and somatic compliant symptoms; however, it was expected that the relationship would be stronger in girls in comparison to boys.
2. It was expected that early pubertal timing would be associated with higher rates of anxious/depressed symptoms, withdrawn/depressed symptoms, and somatic complaint symptoms, in comparison to on-time or late-maturing youth. Again, it was expected that this relationship would be stronger in girls in comparison to boys.
3. It was expected that girls would indicate higher levels of internalizing symptoms, in comparison to boys, namely anxious/depressed symptoms, withdrawn/depressive symptoms, and somatic complaint symptoms.

## Methods

### Procedure

Data was collected in N = 1,342 early adolescents (665 girls; *M*_age_ = 13.26 years, *SD* = 1.27; 677 boys *M*_age_ = 13.19 years, SD = 1.31). The study was reviewed and approved by the Ministry of Higher Education of Kosovo; each school principal from the seven largest municipalities had to review and decide on participation. At participating schools, parents were sent a consent document, informing them of the study and requesting their consent for their child to participate in the study. Consent forms were returned to the schools. Of approximately 2,000 consent documents, 1,478 were returned. The remainder were missing either due to parental refusal to participate in the research, or due to failure to return the consent forms to school. Thus, the total recruited sample included 1,478 early adolescents: due to missing data, the final study sample consistent of N = 1,342 adolescents. Data were collected in school classrooms of each municipality, between November 2019, and March 2020. Participants completed an assent document prior to participation; next, they completed an anonymous self-report paper and pencil survey during school hours, which lasted approximately 40 min. The study followed all ethical guidelines of Helsinki declaration of 1975, as revised in 2008.

## Sample

Participants were 11 years old (13.7%), 12 years 14.2%), 13 years (25.4%), 14 years (32.0%), and 15 + years old (14.8%). The sample included 6th (13.7%), 7th 14.2%), 8th (25.4%), and 9th grade students (46.7%) who were of ethnic Albanian (95.6%) as well other ethnic groups living in Kosovo (4.4%). Final percentage sample divided regionally, was from Peja (9.2%), Prizren (14.7%), Malisheve (12.4%), Mitrovice (17.9%), Gjilan (11.8%), Fushe Kosove (12%), and Gjakove (22.0%),

## Measures

### Demographics Variables

Study variables included age, sex, municipality, socioeconomic status (SES), grade, and ethnicity.

#### **SES**

Parental employment was used to assess family socioeconomic status (SES), based both on mother’s and a father’s employment, ranging from 1 = ” owner/professional official/high degree”, 2 = ” small business owner/professional/IT/ large farm owner/military officer’’, 3 = ” semi-professional worker/skilled craftsman’’, 4 = ”cleric staff sales representatives/artist/other military personnel’’, 5 = ”machine operator/semi-skilled worker such cook, waiter or janitor’’, and 6 = ” laborer or service worker”, and 7 = “unemployed”. Responses were reversed coded and then averaged, so that the higher scores indicated higher family SES.

#### **Pubertal Developmental Scale**

Puberty was assessed by using Pubertal Developmental Scale (Petersen et al., 1988). The self-report scale consists of 5 items, which focus on the development of secondary sexual characteristics. The first three items ask about body growth in height, pubic hair and skin changes, which are for both sexes. Then it is followed by the items for facial hair, deepening of the voice for boys only, and breast development and menarche for girls only. Except menarche question (a dichotomy), the five pubertal items use 4 rating points Likert scale starting from 1 there is no development, 2 developments have barely begun, 3 development was definitely underway, and 4 development was already completed. The girls were asked also to indicate whether they experienced menarche or not. Those who experienced menarche, reported it in months and in years. The measure was internally consistent (girls α = 0.67; boys α = 0.74), indicating acceptable reliability. The pubertal development score was computed by summing across the five items to obtain a total score; the sum of the scores on the five indicators was divided by five in order to preserve the original (1–4) metric.

#### **Body Mass Index**

Body Mass Index (BMI) was calculated using weight in kg and height in m reported by participants: BMI = weight (kg)/ [height (m)]^2^.

#### **Youth Self Report (YSR)**

Participants completed the YSR instrument, consisting of 112 items (Achenbach & Rescorla, 2007). However, the present study only focused on internalizing problems, namely the *withdrawn/depressed subscale* (8 items; sample item: ‘*I am unhappy, sad, or depressed’*), the *anxious/depressed subscale* (13 items; sample item: ‘*I cry a lot’*), and the *somatic compliants subscale* (10 items, sample item: ‘*I have nightmares’*). The anxious/withdrawn item *‘I think about killing myself’* was excluded from the assessment, due to being sensitive question for young participants. The Albanian version was used with permission from official ASEBA’s package representatives for Kosovo (Shahini et al., 2015). The participants responded to the items on a 3-point Likert type scale, by choosing 3 options, (0) *not true at all* (1), *somewhat true*, and *very true* (2) in a six-month lapse. The internalizing broad-band measurement was internally consistent among girls (internalizing broad-band α = 0.81; anxious/depressed α = 0.81; withdrawn/depressed α = 0.61; and somatic complaints α = 0.75 respectively) and boys (internalizing broad-band scales α = 0.79; anxious/depressed α = 0.69; withdrawn/depressed α = 0.45 and somatic complaints α = 0.70, respectively). The Youth Self Report has been established as a reliable instrument for assessing internalizing problems among Kosovar youths (see Shahini et al., 2015); its validity and reliability have been well documented by Achenbach and Rescorla (2007). Fairly significant agreement between adolescent self-reports and parent reports in internalizing symptoms have been reported (Thomas et al., 1990).

## Plan of Analysis

Five pubertal categories were developed based on Petersen and colleagues (1988): prepubertal, beginning of pubertal, mid pubertal, advanced pubertal and post pubertal. Classification into one of five pubertal categories (prepubertal, beginning of pubertal, mid pubertal, advanced pubertal and post pubertal) is based on the level of development reported on the three indicators of the pubertal changes thought to be most salient for each sex. For girls this include: pubic hair growth, breast development and menarche, whereas, for boys, they are: development of pubic hair, facial hair and voice changes. For girls, category *prepubertal* (1) is assigned if girls report no development in any of the three indicators (i.e. received a combined score of ‘3’ for the three indicators).** (**2) *beginning of pubertal*: if girls report no menarche along with some development of either breast or pubic hair but not both of them (i.e. a combined score of ‘3’ for the last two indicators). (3) *midpubertal:* if girls report no menarche along with some development of either breast and pubic hair or more development on at least one of these characteristics (i.e. a combined score of ‘4’ or more for more breast and body hair). (4) *Advanced pubertal:* if girls report menarche in combination with less than complete breast and/or pubic hair (i.e. a combined score of ‘7’ or less on these two indicators).** (**5) *Post pubertal*: if girls report menarche along with completed development for both pubic hair and breast (e.g. a combined score of ‘8’ on these two indicators).

For boys, (1) *prepubertal category* is assigned if boys report no development on any of these three characteristics (a combined score of ‘3’). (2) *Beginning of pubertal*: if boys report initial development on one or two characteristics, or more development in one characteristic but no development on the other two (i.e. a combined score of ‘4’ or ‘5’). (3) *mid-pubertal*: if boys report beginning development on all three or advanced development on one or two combined with little or no development on the others (i.e. a combined score of ‘6’ or ‘7’ or ‘8’). (4) *advanced pubertal*: if they report advanced development on all three or beginning development on one combined with advanced or completed development on another or completed development on the third (e.g., a combined score of ‘9’ or ‘10’ or ‘11’). (5) *post pubertal*: if boys report completed development of pubic hair, facial hair, and voice (i.e., a combined score of ‘12’).

To determine early, on time and late categorization, first, scores of the pubertal development scale were standardized (changing into the distribution with a mean of 0, and standard deviation of 1) for each age: 11, 12, 13, 14, and 15 (few cases of 16 were also collapsed with the age 15 group). Then, early was defined to be having pubertal development scores greater than 1 (1); on time = scores between -1 and 1 (2); and late = scores below -1 (3) (see Flannery et al., 1993; Steinberg, 1987).

The first step of the analyses was to calculate descriptive statistics of the study variables for boys and girls separately, followed by computation of bivariate Pearson’s correlations. Next, a series of Ordinary Least Squares (OLS) regressions were run to test the study hypotheses. Due to high correlations between pubertal timing and Pubertal Development, their effects were tested in separate regressions. The following regressions were tested for boys and girls separately: 1) where the dependent variable was anxious/depressed symptoms, 2) with withdrawn/depressed symptoms as the dependent variable, and 3) somatic complaints being the dependent variable. First step of each regression included family socioeconomic status as the independent variable, then in the next step, BMI, and pubertal timing (or alternatively, pubertal development) were added. Analyses were conducted using SPSS 26.

## Results

Descriptive statistics of the study variables are reported in Table 1. The average age of menarche in girls was 12.35 years. T-tests were conducted to test mean-level differences in each type of internalizing symptoms/domains by sex. Findings from these tests indicated that there were significantly higher levels of internalizing symptoms among girls compared to boys (t = -12.46, *p* < 0.001, for Anxious/depressed; t = -10.40, *p* < 0.001, for Withdrawn/depressed; and t = -7.17, *p* < 0.001, for Somatic complaints).

**Table 1:** Means and Standard Deviations of Study Variables for Boys and Girls

	Mean	SD	Min; Max
**Girls** (N = 665)			
Age	13.26	1.27	11; 16
Family SES (parental education)	3.59	1.68	1; 7
BMI	19.76	3.52	8.15; 40.97
*Pubertal indices:*			
1. Growth spurt	2.76	0.70	1; 4
2. Body hair (underarm and pubic hair)	2.62	0.88	1; 4
3. Breast development	2.58	0.70	1; 4
4. Skin change	2.10	0.85	1; 4
5. Menarche (% yes)	71%		0; 1
Pubertal Development (alpha = 0.67)	2.63	0.62	0.80; 4
*Pubertal timing:*			
1. Early (%)	17.8		
2. On time (%)	68.4		
3. Late (%)	13.8		
*Internalizing symptoms:*			
1. Anxious/depressed (12 items, alpha = 0.81)	0.55	0.38	0; 1.92
2. Withdrawn/depressed (8 items, alpha = 0.61)	0.63	0.36	0; 2
3. Somatic complaints (10 items, alpha = 0.75)	0.37	0.32	0; 1.80
**Boys** (N = 677)			
Age	13.19	1.31	11; 16
Family SES (parental education)	3.59	1.71	1; 7
BMI	20.74	4.56	9.23; 51.88
*Pubertal indices:*			
1. Growth spurt	2.57	0.81	1; 4
2. Body hair (underarm and pubic hair)	2.57	0.76	1; 4
3. Facial hair	1.76	0.72	1; 4
4. Skin change	2.03	0.88	1; 4
5. Deepening of the voice	2.39	0.87	1; 4
Pubertal Development (alpha = 0.74)	2.26	0.57	1; 3.80
*Pubertal timing:*			
1. Early (%)	16.1		
2. On time (%)	65.2		
3. Late (%)	18.6		
*Internalizing symptoms:*			
1. Anxious/depressed (12 items, alpha = 0.69)	0.32	0.26	0; 1.58
2. Withdrawn/depressed (8 items, alpha = 0.45)	0.44	0.28	0; 1.63
3. Somatic complaints (10 items, alpha = 0.70)	0.25	0.26	0; 1.70

Alpha refers to Cronbach’s alpha

Table 2 presents bivariate Pearson’s correlations of the study variables. Family SES was positively associated with the total pubertal development status/stage in girls as well as in the boys (*r* = 0.10, *p* = 0.041), however, it was unrelated to any of the three internalizing symptoms/outcomes. Despite this fact, the effect of family SES was controlled for, in subsequent regressions. Being an early matured compared to late (but not on time) was related to higher BMI for both sexes (*r* = 0.30, *p* < 0.001). Being an early mature compared to late was also related to higher rates of anxious/depressed, and somatic complaints among girls (*r* = 0.24 and 0.22. respectively, *p* < 0.001) and with withdrawn/depressed in boys (*r* = 0.13, *p* = 0.023). Being *early* compared to *on-time* was related to only somatic complaints in girls, however to much lesser extent (*r* = 0.09, *p* = 0.042) than it was compared to *late*; and in boys, it was related to withdrawn/depressed symptoms (*r* = 0.09, *p* = 0.032). Pubertal development status/stage was positively associated with each internalizing symptom in girls (*r* = 0.31, 0.26, 0.22, *p* < 0.001, for anxious/depressed, withdrawn/depressed, and somatic complaints, respectively) but only with withdrawn/depressed among boys (*r* = 0.08, *p* = 0.043). As expected, all the internalizing symptoms were positively and highly correlated with each other in both sexes.

**Table 2:** Correlations of Study Variables by sex

**A. Girls**								
*Variables*	1	2	3	4	5	6	7	8
1. Age								
2. Family SES	.04							
3. BMI	.23***	.04						
4. Pubertal development	.62***	.10*	.28***					
5. Timing: Early1	-.04	.05	.02	.51***				
6. Timing: Early2	.04	.09	.30***	.87***				
7. Anxious/depressed	.30***	.00	.09*	.31***	.07	.24***		
8. Withdrawn/depressed	.31***	-.01	.15***	.26***	.03	.09	.69***	
9. Somatic complaints	.17***	.01	.06	.22***	.09*	.22***	.62***	.51***

**B. Boys**								
*Variables*	1	2	3	4	5	6	7	8
1. Age								
2. Family SES	.06							
3. BMI	.08*	.08						
4. Pubertal development	.41***	.10*	.18***					
5. Timing: Early1	.04	.04	.04	.65***				
6. Timing: Early2	.07	.05	.30***	.92***				
7. Anxious/depressed	.08*	.03	.06	.04	-.05	.04		
8. Withdrawn/depressed	.15***	-.01	.04	.08*	.09*	.13*	.50***	
9. Somatic complaints	.05	.03	.02	.04	-.03	.06	.54***	.37***

Timing = pubertal timing; reference category for pubertal timing, for “Early1” is on time, whereas for “Early2” is late

*p < .05; **p < .01; ***p < .001

Table 3 reports results from regression analyses predicting internalizing symptoms by BMI, pubertal timing, and pubertal development status/stage, with family SES used as a control variable. Due to high correlations between pubertal timing and pubertal development, their effects are tested in separate regressions. The effects of pubertal timing are modeled using dummy-coded variables, one representing the coefficients of *on-time* and the other *late* groups compared to *early* group*.* For girls, the results showed that being late in maturation in comparison to early was negatively associated with anxious/depressed symptoms as well as somatic complaint symptoms (β = -0.17, for both). Being *on-time* in comparison to *early* was also positively associated with somatic symptoms (β = -0.10). BMI was unrelated with these two internalizing symptoms controlling for pubertal timing; however, it was positively associated with withdrawn/depressed symptoms (β = 0.15); this positive relationship was also found in the second regression, controlling for the effect of pubertal development status/stage (β = 0.08). Pubertal development status/stage of maturation was significantly and positively associated with each measure of internalizing symptoms among girls, controlling for BMI and family SES (βs = 0.31, 0.24, and 0.22, respectively). Overall, the model explained 9.8% of variance in anxious/depressed symptoms, 7.4% in withdrawn/depressed symptoms, and 4.9% in somatic complaint symptoms.

**Table 3:** Regression analyses for internalizing symptoms predicted by SES, BMI, pubertal timing, and pubertal development status by sex

**Girls**									
*A. Pubertal timing*									
Predictors	1. Anxious/depressed			2. Withdrawn/depressed			3. Somatic complaints		
	β	SE	p	β	SE	p	β	SE	p
*Step1:*									
Family SES	0.01	0.01	.913	-0.01	0.01	.836	0.01	0.01	.789
*Step 2:*									
BMI	0.06	0.01	.108	0.15	0.01	< .001	0.04	0.01	.375
Pub. timing: On time	-0.08	0.04	.097	-0.03	0.04	.561	-0.10	0.04	.042
Pub. timing: Late	-0.17	0.06	.001	-0.04	0.06	.413	-0.17	0.05	.001
R^2^	0.026			0.024			0.021		

*B. Pubertal development*									
Predictors	1. Anxious/depressed			2. Withdrawn/depressed			3. Somatic complaints		
	Β	SE	p	β	SE	P	β	SE	p
*Step1:*									
Family SES	0.01	0.01	.913	-0.01	0.01	.836	0.01	0.01	.789
*Step 2:*									
BMI	-0.01	0.01	.976	0.08	0.01	.041	-0.01	0.01	.863
Pubertal development	0.31	0.01	< .001	0.24	0.01	< .001	0.22	0.01	< .001
R^2^	0.098			0.074			0.049		

**Boys**									
*A. Pubertal timing*									
Predictors	1. Anxious/depressed			2. Withdrawn/depressed			3. Somatic complaints		
	β	SE	p	β	SE	p	β	SE	p
*Step1:*									
Family SES	0.03	0.01	.442	-0.01	0.01	.861	0.03	0.01	.451
*Step 2:*									
BMI	0.05	0.01	.218	0.03	0.01	.507	0.01	0.01	.880
Pub. timing: On time	0.06	0.03	.269	-0.10	0.03	.062	0.04	0.03	.465
Pub timing: Late	-0.02	0.04	.716	-0.10	0.04	.070	-0.04	0.04	.442
R^2^	0.011			0.001			0.007		

*B. Pubertal development*									
Predictors	1. Anxious/depressed			2. Withdrawn/depressed			3. Somatic complaints		
	β	SE	p	β	SE	p	β	SE	p
*Step1:*									
Family SES	0.03	0.01	.442	-0.01	0.01	.861	0.03	0.01	.451
*Step 2:*									
BMI	0.05	0.01	.206	0.01	0.01	.731	0.01	0.01	.831
Pubertal development	0.04	0.01	.328	0.13	0.01	.002	0.05	0.01	.274
R^2^	0.006			0.019			0.003		

Reference category for pubertal timing variables is “early”

*p < .05; **p < .01; ***p < .001

For boys, regression analysis results provided evidence that there were no significant associations with either BMI or pubertal timing; the only significant relationship found was for pubertal development status/stage of maturation which was positively with associated withdrawn/depressed symptoms (β = 0.13). This model explained 1.9% of variance, the largest across these six different models.

## Discussion

The present provides evidence that pubertal status/stage was positively associated with rates of anxious/depressed symptoms, withdrawn/depressed symptoms, and somatic complaint symptoms in girls; it was also positively associated with rates of withdrawn/depressive symptoms only in boys. In addition, the evidence indicated that early maturational timing was positively associated with anxious/depressed and somatic complaint symptoms in girls, but not in boys.,

Overall the rates of internalizing symptoms (independent of status/stage and maturational timing) of anxious/depressed symptoms, withdrawn/depressed symptoms, and somatic complaint symptoms appeared higher among girls in comparison to boys, consistent with previous work that was carried out in the same developmental context (Fanaj et al., 2015; Shahini et al., 2015), but also with work from other contexts (Ge et al., 1994; Hankin et al., 1998). Early maturing girls with adult-like features oftentimes are exposed to societal pressure on how they would be expected to behave at a certain age, which would make them prone to emotional problems due to the asynchrony between physical and cognitive development (Natsuaki et al., 2015). Many societies would expect that early maturing boy’s appearance might be an advantage of male physical development, therefore advanced pubertal development status among boys, and early timing would predict fewer emotional problems. In fact, accelerated pubertal development might be considered positive and elicit positive responses from peers and friends.

The beginning of adolescence is marked by an increase pressure and demand for conformity to cultural gender norms from peers, parents or others (see Hill & Lynch, 1983, gender intensification hypothesis). Kosovar families still follow patriarchal values related to family roles (e.g., females responsible for childbearing, and household while men would be dominant and be held responsible for incomes for instance) (Latifi, 2014). Traditional gender roles (feminine roles) which are linked to some extent with patriarchal values, have been found to be associated with adolescent poor mental health, particularly girls’ anxiety, for instance (Aparicio-García et al., 2018). The mean age of menarche was 12.35 years in the present study; this was slightly younger than the one reported by Boshnjaku and colleagues, which was 13.5 years (Boshnjaku et al., 2016), but also similar to reports from neighboring countries such Montenegro, 12.15 years (Šćepanović et al., 2019) or reports on girls from the United States, 12.25 years (Biro et al., 2018).

Consistent with the first study hypotheses, based on correlations advanced pubertal status/stage was positively associated with anxious/depressed symptoms, withdrawn/depressed symptoms, and somatic complaint symptoms in girls, but only with withdrawn/depressive symptoms in boys. These findings were also made in regression analyses for girls, but not for boys. These findings about girls namely that advanced pubertal status was positively associated with anxiety and depressive symptoms was consistent with previous work (Ge et al., 2001; Angold et al., 1998; Lewis et al., 2018; Reardon et al., 2009, Huerta & Brizuela-Gamiño 2002; Deardorff et al., 2007; Conley & Rudolph, 2009); the findings for boys was also consistent with previous evidence (Ge et al., 2001, 2006; Richardson et al., 2006). In fact, Conley and Rudolph (2009) also found no associations between advanced pubertal stage and depressive symptoms in boys. The evidence appears to support the *pubertal status hypothesis* which suggests that advancing through puberty (independent of pubertal onset/timing) places girls at risk for depressive symptoms (Lewis et al., 2018). These effects are thought to be driven by several hormone levels that surge during adrenarche as well as psychosocial mechanisms. First, it has been suggested that there is peak in adolescent female depressive symptoms in mid adolescence around ages 12 or 13 years (Angold et al., 1998; Ge et al., 1994; Hankin et al., 1998). Ge et al. (1994) found evidence that depressive symptoms in girls covaried with stressful life events. Maturational changes and puberty might increase a girl’s susceptibility to stressful life events and negatively affect their affective state, including depression (Ge et al., 2001). Girls might also be at greater risk for rumination than boys (e.g., see the Response Style Theory by Nolen-Hoeksema, 1991), which might in part explain sex differences in internalizing symptom rates. In addition, poor emotional control and high levels of family conflict have been reported to be predicting the in rates of depressive symptoms in girls (Patton et al., 2008). Finally, Graber and colleagues (2006) found evidence that estradiol levels in girls are associated with risk for depressive symptoms (breast development is largely controlled by estradiol levels; Lewis et al., 2018).

Early maturational/pubertal timing was positively associated with anxious/depressed symptoms and somatic complaint symptoms in girls only, in comparison to late maturing girls. This partially supported the second study hypothesis. On-time pubertal timing was also associated with fewer somatic complaint symptoms in comparison with early maturing girls. The evidence from the present study is consistent with previous research which found a positive association between pubertal timing and internalizing symptoms in girls, but not boys (Blumenthal et al., 2011; Conley & Rudolph, 2009; Graber et al., 1997). The evidence was also consistent with previous work which found a positive link between early pubertal timing and somatic complaint symptoms in girls (Rhee, 2005; see also Kløven et al., 2017). A higher incidence of somatic complaint symptoms in girls than boys, might be attributed to some extent to the interplay between stress and hormonal changes being more evident in girls (Williams & Zahka, 2017), or particular hormones in girls. Another potential explanation includes that some particular physical symptoms, such as headaches, are more prevalent among early maturing girls, while musculoskeletal pain is more common among boys (Rhee, 2005). Our findings support the *developmental readiness hypothesis* (stage termination hypothesis) assertation, that early maturational timing posits greater risk for emotional adjustment problems, particularly in girls.

Navigating through puberty is a new challenge for adolescents, due to its rapid physical changes, changes that are manifested by emotional and psychosocial changes in adolescent girls and boys. Pubertal development in fact seems to exacerbate the observed physical and cognitive asynchrony in youth. This maturational disparity hinders emotional adjustment due to adolescents simply not being well prepared to adjust to both the profound cognitive and physical changes, consistent with the maturational disparity hypothesis (Ge & Natsuaki, 2009) and the developmental readiness hypothesis (Negriff & Susman, 2011). As a result, early puberty contributes to elevated levels of affective problems during early adolescence. Next, it is possible that the early pubertal timing-internalizing problems relationship unfolds due to girls’ individual vulnerabilities, including low self-regulation skills, for instance (Crockett et al., 2013). Likewise, levels of anxiety, depression, and psychological stress appear to be higher among girls than boys, due to their dissatisfaction with their social contacts and poor social support (Van Droogenbroeck et al., 2018). Girls appear to be susceptible to peer pressure during puberty, which might indirectly contribute to the present study findings. Conley and Rudolph (2009) found a positive association between early pubertal timing and heightened risk for depression in girls exposed to high levels of peer stress. In this sense, growing up in the Kosovar patriarchal culture might have contributed to some of the observed sex differences; culturally, accelerated pubertal development in boys is almost an expectation, while for girls accelerated bodily changes are a liability thereby contributing to increased internalizing symptoms.

This view has been elaborated by Hill and Lynch (1983), namely the intensification of the gender role expectations hypothesis, which maintains that both boys and girls experience increased pressures to conform to culturally sanctioned gender roles. Such expectations might partially address some of the observed differences in the present study. Next, an additional explanation might include that four genetically vulnerable girls, normal hormonal cycling might trigger dysregulation of neurotransmitter systems, leading to increases in depressive symptoms (Nolen-Hoeksema & Hilt, 2009). Therefore, the interactions between hormonal changes and psychosocial experiences increases the likelihood on elevated depressive symptoms, more so among girls than boys (Frank & Young, 2000). That is, much of the adolescent emotional adjustment depends on the cognitive, hormonal, and social reaction following the early onset of puberty, thus foretelling either a positive or negative emotional development. In addition, the ongoing economic and social transformations in the Kosovar contexts, defined by ongoing collective trauma, might also have contributed to study findings. Ge and colleagues (2001) found that when early maturation was accompanied by recent stressful life events, this placed girls at higher risk for developing depressive symptoms. Considering that collective trauma and rapid societal changes in Kosovo have occurred over the past two decades, these stressful life events might also have indirectly affected girls’ levels of internalizing symptoms. Finally, no association was found between SES and pubertal timing in the present study; evidence from other developmental contexts, however, has provided evidence that youth with lower SES is associated with earlier breast development (thelarche) and a longer duration of puberty (Oelkers et al., 2021).

## Conclusion

The current study findings provided evidence that advanced pubertal development status/stage was positively associated with internalizing problems in girls, namely anxious/depressed symptoms, withdrawn/depressed symptoms, and somatic complaint symptoms, but only with withdrawn/depressed symptoms in boys (correlations only, not based on regression findings). The results also showed that early maturing girls scored higher on measures of anxious/depressed symptoms, and somatic complaint symptoms, in comparison to on-time or late maturing girls. Thus, early maturation poses a risk for affective problems in mostly for girls in the present study. The rates of unemployment in Kosovo, ongoing societal transitions from a collectivist culture to a more individualistic one, as well as direct or indirect chronic exposure to stressful life events for adolescents, are likely to have impacted study findings (see Ge & Natsuaki, 2009; Ge et al., 2001).

The study results support the pubertal stage hypothesis (Lewis et al., 2018) which posits that advancing through puberty increases the risk of internalizing problems in adolescents, particularly for girls. The results were also consistent with the developmental readiness hypothesis (Negriff & Susman, 2011; Petersen & Crockett, 1985), which holds that early maturational timing, particularly for girls, heightens the risk of internalizing problems, in comparison with on time or late maturing youth. Finally, based on the contextual amplification hypothesis (Ge & Natsuaki, 2009), it was expected that experiencing puberty in a high risk context (e.g., low SES, experiences and history of ethnic violence, following war trauma and intergenerational trauma), would further exacerbate risk for internalizing problems, in particular.

The present study leads to the following main conclusions: (1) pubertal status/stage plays an important role in the development of internalizing problems among Kosovar youth, particularly for girls; (2) early pubertal timing was identified as a risk factor for internalizing symptoms in girls, but not boys; (3) on-time and late maturation seem to function as a protective mechanism for Kosovar girls; (4) internalizing problems appeared to be more prevalent among girls than boys; and (5) the unique contextual characteristics of Kosovo, defined by relatively low socioeconomic status, a history of ethnic tensions and violence, and a history of war as well as subsequent trauma, particularly among parents or grandparents, might have indirectly impacted the emotional well-being among youth, and therefore, placed them at greater risk for internalizing problems.

## Limitations

The present study contributes to an understanding of the role of early pubertal development on internalizing problems risk, however, a number of limitations should be noted. Due to cross-sectional nature of the study, it cannot provide evidence of causality; in other words, the study cannot contribute to an understanding about developmental changes or processes in either physical development or internalizing problems, or about the direction of the effects. In this sense, the study is very limited in its ability to provide information about the sequence of pubertal events (whether breast development precedes pubic hair, and vice versa), or pubertal tempo (navigating puberty slower or faster than their counterparts), both associated with internalizing problems.

A further study limitation includes the sole reliance on self-reports which therefore introduces the potential for monomethod bias. In other words, the observed relationships might be inflated due to shared method variance. Future work needs to employ multiple informants such measuring the pubertal development indices from a trained medical team, or also assessment of the hormone levels that contribute to adolescent sexual maturation during early phase of puberty, in addition to the self-report measures of pubertal development. At the same time, a moderate amount of agreement has been found between adolescent self-reports and parent ratings measuring internalizing symptoms (e.g., Neill, 2016; Wang et al., 2014). Future work should be longitudinal in nature as well as include multiple informants, to overcome mono-method bias.

Another study limitation is the use of Body Mass Index to measure obesity. Previous work has shown that BMI does not always capture excess body fat, also related to different body types and sizes (Nuttall, 2015; Romero-Corral et al., 2008).

A further limitation concerns low reliabilities of the withdraw/depressed subscale in both female and male participants. However, similar size estimates have been found (0.52–0.64) in previous work (Verhulst et al., 2003); boys withdrawn/depressed symptom scale (0.54) and girls (0.62; Geibel et al., 2016). Despite the fact that these scales had low reliability, based on construct and face validity considerations, a decision was made to use them in the present investigation. A low reliability simply indicates that the observed associations among constructs might attenuated due to low reliability of some of the measures, or in other words, the actual associations might be slightly larger given higher reliability estimates.

It is also not possible to comment about the extent to which the effects of early pubertal timing and pubertal status/stage persist developmentally into late adolescence, or early adulthood, or whether they wane with age. Next, the study was based on a large convenience sample of Kosovar youth; this means that the sample was not representative of Kosovar youth more generally speaking. Due to the large sample size and the focus on an understudied Kosovar adolescent population, these unique features might in part at least outweigh important study limitations. Future work would need to include a representative sample of ethnic Albanians and other ethnic youth (e.g., Roma youth) living in Kosovo to observe the extent to which ethnicity might impact pubertal development, internalizing problems as well as the relationship between the two. It is also important to note that the present investigation did not test a number of known correlates of puberty as well as internalizing problems nor did it test competing, alternative study hypotheses It is possible, thus that the early onset of internalizing problems would accelerate the onset of BMI and physical development among youth, and the early puberty and internalizing problems link might additionally be moderated by other variables, such as low parental attachment, for instance. Finally, despite these limitations, the study fills an important gap about scholarship focused on adolescence from Kosovo.

## Contribution and Implication for Practice

The study findings point to several important practical implications, informing policy making and decisions as well as program development and delivery that facilitate positive mental health and wellbeing. Early pubertal timing was found to be a correlate of internalizing symptoms among girls; in addition, advanced pubertal status (and not pubertal timing) was positively associated with withdrawn/depressive symptoms among boys. These are novel observations in the Kosovar context, which identifies risk related to early pubertal development and associated adjustment problems. With emotional problems peaking in mid-adolescence (Hankin et al., 1998; Lewis et al., 2018), psychological therapeutic interventions should target youth during middle adolescence. More specifically, psychological services might target and provide support to girls that present features of early puberty (adult-like morphological body features, or rapid progress through puberty), exhibit body discomfort, or even are victims of bullying due to appearance changes tied to puberty.

Study findings also potentially inform school policies and programs; these might include psychoeducational interventions focused on sexual and reproductive health (sex education curricula), to better inform adolescents about their expected bodily changes. Previous work has shown that when adolescents have prior knowledge about their expected bodily changes, they are less likely to develop negative attitudes about them (Belgrave, 2009), and thus, potentially also less likely to feel anxious or depressed about them. The Ministry of Education of Kosovo, the Kosovo Institute of Public Health, with support by the United Nation Population Fund (UNFPA), has provided updated manuals about adolescent sexual and reproductive health to teachers (see Begolli et al., 2023; Berisha et al., 2021). In fact, a recent meta-analysis has shown that digital evidence-based mental health programs for adolescents might help relieve anxiety, enhance protective factors, and promote well being among youth (Wright et al., 2023). Such programs can be delivered at relatively low cost, making this a very tangible approach to support youth in LMICs (low- and middle-income countries).

Beyond schools, the present study findings also provide some potential recommendations for parents. Parents of early maturing girls might consider improving their relationship with their teenager, specifically focused on improving both closeness and support. Previous work has shown that when mothers increase their support towards their early maturing daughters, this contributes to down regulating negative emotions and up regulating positive ones (Lougheed et al., 2016).

Finally, future work should consider studying more diverse samples including understudied groups in Kosovo (e.g., Roma and other ethnic minority groups groups), to better understand pubertal experiences among all adolescents.

## Data Availability

The datasets generated during and/or analyzed during the current study are available from the corresponding author, on reasonable request.
